# The Optimal Emergency Department Management of Out‐of‐Hospital Supraglottic Airways

**DOI:** 10.1111/acem.70171

**Published:** 2025-10-24

**Authors:** Aaron E. Robinson, Matthew E. Prekker, Marc L. Martel, Brian E. Driver

**Affiliations:** ^1^ Department of Emergency Medicine Hennepin County Medical Center Minneapolis Minnesota USA; ^2^ Department of Emergency Medicine University of Minnesota Minneapolis Minnesota USA; ^3^ Hennepin EMS Hennepin Healthcare Minneapolis Minnesota USA; ^4^ Division of Pulmonary and Critical Care Medicine, Department of Medicine Hennepin Healthcare Minneapolis Minnesota USA

Every emergency physician has managed a critically ill patient who arrived by ambulance ventilated with a supraglottic airway (SGA) placed by prehospital clinicians. On the one hand, patients usually receive robust oxygenation and ventilation through these devices [1, 2], while on the other hand, they are not a definitive airway and the emergency physician must consider next steps: Should the SGA be removed for intubation? Kept in place while other resuscitation interventions are prioritized or used as a conduit for the endotracheal tube? Deferred to another clinician? In this editorial, prompted by correspondence by Braude et al. and Trostel et al. in this issue, we outline practical answers to these questions.

Three principles should guide management. First, most patients require a cuffed endotracheal tube before leaving the ED, barring unusual circumstances—but there is rarely a need to rush [3]. Second, the simplest and safest approach is preferred; avoid unnecessary complexity. Third, techniques should rely on tools familiar to emergency physicians and widely available in the ED.

## 1 Initial Assessment

The first task is to confirm whether the SGA is providing adequate ventilation. End‐tidal capnography (preferred), oxygen saturation, thoracic ultrasound, auscultation, and visual inspection of chest rise can all be used for this purpose. If ventilation is inadequate despite troubleshooting, the SGA should be removed immediately and tracheal intubation attempted. Alternatively, a different model or size of SGA could be placed. Copious vomiting or regurgitation of stomach contents also mandates SGA removal.

If the SGA is functioning, clinicians have time to address other resuscitation priorities before definitive airway management is performed. Intubation typically occurs within 30 min of ED arrival but may be delayed if clinically necessary [3]. Meanwhile, the SGA is generally an excellent preoxygenation device, particularly in patients who may be difficult to ventilate with a mask (e.g., beard, facial trauma). When an SGA is left in place, neuromuscular blockade and sedation should be administered as SGAs stimulate periglottic nerves and may induce vomiting [4]. Neuromuscular blockade also improves ventilation quality, reduces oxygen consumption, and readies the patient for eventual laryngoscopy.

## 2 Default Approach: Remove and Intubate

In the vast majority of patients, the best course is to remove the SGA and proceed with rapid sequence intubation using video laryngoscopy. This approach is highly successful [5, 6], and if any difficulty is encountered, the patient may be easily rescued with bag‐mask ventilation or reinsertion of the SGA [7, 8]. We disagree with the unreferenced assertion made by Trostel et al. that removing an SGA is a “high risk” maneuver [9]. When the SGA is reinserted, we recommend using an SGA that can be intubated through and, ideally, has a separate lumen that exits at the esophageal inlet to facilitate orogastric tube placement and gastric decompression. These capabilities are found on the newer‐generation SGAs outlined in Table 1.

**TABLE 1:** Overview of Common Supraglottic Airway Types.

LMA type	Manufacturer	Max size ETT	Can intubate through	Gastric port	Adjustable cuff
LMA Fastrach	Teleflex, Athlone, Ireland	8.0	Yes	No	Yes
AirQ3G	Cookgas Medical, Bloomington, Indiana, USA	8.0	Yes	Yes	Yes
iGel	Intersurgical, Wokingham, Berkshire, UK	7.0	Yes	Yes	No
Auragain	Ambu, Ballerup, Denmark	7.5	Yes	Yes	Yes
LMA Supreme	Teleflex, Athlone, Ireland	6.0	Yes	Yes	Yes
King LTS‐D	Ambu, Ballerup, Denmark	N/A	No	Yes	Yes

If EMS attempts at orotracheal intubation failed leading to prehospital SGA placement, the default strategy of SGA removal followed immediately by orotracheal intubation is still reasonable. Prehospital intubation failure does not translate to an equivalent likelihood of failure in the ED [10]. Emergency physicians should anticipate a more difficult airway in such cases and prepare accordingly, but this is not a convincing reason to avoid SGA removal. We previously reported our experience with leaving certain SGAs (e.g., King laryngeal tubes and specific laryngeal mask airways) in place during video laryngoscopy attempts, thereby facilitating more rapid re‐ventilation should the attempt fail [11], but believe this technique is unnecessarily complicated. A simpler, safer option is to remove the SGA, attempt intubation, and then reinsert the SGA if needed.

## 3 Less Common Approach: Intubate Through the SGA

In rare cases of severe anatomic distortion—such as malignancy, trauma, or angioedema of the head and neck—it is reasonable to attempt intubation through the existing SGA, if the SGA permits intubation through it. We should emphasize that most patients with structural distortion of the upper airway who are ventilated effectively through an SGA can still be managed by removing the SGA, attempting intubation, and reinserting the device if the attempt fails. There is no evidence that a previously functioning SGA will abruptly fail to ventilate after reinsertion. Thus, mere suspicion of a difficult intubation is not sufficient reason to necessitate intubation through the SGA.

If intubation through the SGA is pursued, both blind tube passage and endoscope‐guided methods are reasonable options. Blind intubation is straightforward to attempt but less likely to be successful than intubation through an SGA with endoscopic guidance. Blind intubation success rates vary from 50% to 80% depending on the specific SGA [2, 12, 13].

The introduction of single‐use endoscopes into ED practice has improved access to flexible endoscopy for airway procedures. Video laryngoscope manufacturers offer endoscopes of varying sizes that are compatible with the VL monitor. The technique described by Trostel et al. achieved 65% success among 17 patients and involved passing the endoscope through the SGA and advancing it to the carina, then cutting the flexible scope where it meets the handle. Next, the SGA is removed over the cut flexible scope (now functioning as a tracheal tube introducer or bougie), and laryngoscopy and intubation are performed. Braude et al. reported 100% success in 20 patients using a related method: partial insertion of the endotracheal tube into the SGA followed by endoscopy through the tube and positioning the scope deep into the trachea, then advancing the tube and removing both the endoscope and SGA [14]. In both approaches, ventilation is maintained with the use of a 90‐degree swivel elbow connector which also accommodates the endoscope.

## 4 Our Approach at Hennepin County Medical Center Emergency Medicine

The Hennepin County Medical Center ED has been intubating through SGAs for more than 20 years using both blind and endoscopic techniques [2, 15]. We recommend including one or more SGA models with intubation capability in every ED airway cart. Our preferred SGAs accommodate standard sized tubes up to size 8.0 mm, have an inflatable cuff that can be adjusted to optimize the seal around the glottis, and have a gastric port that permits simultaneous passage of a suction catheter into the stomach (Table 1). The Hennepin ED currently has three SGAs on hand for all airway management: the King LTS‐D (Ambu, Ballerup, Denmark), LMA FastTrach (Teleflex, Athlone, Ireland), and AirQ3G (Cookgas Medical, Bloomington, IN). While stocking one SGA is mandatory, we recommend having two styles: one in the LMA family (e.g., AirQ3G) and one in the laryngeal tube family (e.g., King LTS‐D). Anecdotally, there have been instances in our ED where one SGA style failed to provide adequate ventilation while the other type has succeeded.

We intubate via the LMA FastTrach or the AirQ3G at Hennepin with either blind insertion of the endotracheal tube or endoscopic guidance. Typically, gentle blind passage is attempted prior to attempting with the use of an endoscope. Our endoscopic approach is similar to that described by Braude et al. After confirming that the trachea is intubated using the endoscope and waveform capnography, the SGA is removed using a removal stylet that maintains the position of the endotracheal tube while the SGA is removed over it (AirQ Removal Stylet, CookGas Medical, Bloomington, IN, USA).

We train our residents in SGA use in simulation and cadaver labs, and emphasize their use during case‐based discussions at weekly critical care review conferences. We aim to have every graduating resident place an SGA during clinical care at least 3 times, and perform intubation through the device at least once. This goal allows a low threshold for the use of SGAs for preoxygenation and rescue ventilation. This prepares our graduates to confidently manage a failed airway by deploying an SGA [7], thereby stabilizing the patient and allowing for a controlled transition to a surgical airway or subsequent intubation attempts.

Given the increasing use of SGAs in the prehospital setting, emergency physicians must be experts in the post‐arrival management of these patients. While the default approach is to maintain the SGA during initial resuscitation before proceeding with SGA removal and rapid sequence intubation, this is not always optimal or feasible. Therefore, clinicians must also be proficient in securing a definitive airway by intubating directly through the SGA, using both blind and endoscopic techniques. To ensure this critical skill is universal, tracheal intubation through an SGA should be a mandatory component of every emergency medicine residency's airway curriculum.

## Author Contributions

A.E.R., B.E.D., M.E.P., M.L.M. conceived and designed the study. A.E.R., B.E.D., M.E.P., M.L.M. drafted the initial manuscript and made final editorial decisions; all authors contributed substantially to its revision.

## Conflicts of Interest

The authors declare no conflicts of interest.

## Data Availability

Data sharing not applicable to this article as no datasets were generated or analysed during the current study.
